# Oncolytic Adenovirus with SPAG9 shRNA Driven by DD3 Promoter Improved the Efficacy of Docetaxil for Prostate Cancer

**DOI:** 10.1155/2022/7918067

**Published:** 2022-04-30

**Authors:** Meng Lu, Fu-kun Wei, Chuang Wu, Zi-yang Xu, Li-jun Mao, Dong-rong Yang

**Affiliations:** ^1^Department of Urology, The Second Affiliated Hospital of Soochow University, Suzhou 215004, Jiangsu Province, China; ^2^Department of Urology, The Affiliated Hospital of Xuzhou Medical University, Xuzhou 221002, Jiangsu Province, China

## Abstract

Prostate cancer (PCa) is a common malignant tumor of the male urinary system and ranks the second in the causes of tumor-related deaths. Differential display code 3 (DD3) is a noncoding gene that is specifically expressed in PCa. High expression of sperm-associated antigen 9 (SPAG9) is closely related to tumorigenesis of PCa, and SPAG9 is a therapeutic target for PCa. In this study, a new oncolytic adenovirus DD3-ZD55-SPAG9 was constructed by using DD3 promoter to enhance the efficacy and safety of adenovirus. The combined use of DD3-ZD55-SPAG9 and docetaxel showed that DD3-ZD55-SPAG9 significantly improved the anti-tumor efficacy of docetaxel in PCa both in vitro and in vivo. The mechanism was related to the induction of tumor cell apoptosis and the inhibition of tumor cell invasion. In conclusion, DD3-ZD55-SPAG9 combined with docetaxel is an effective strategy for PCa therapy.

## 1. Introduction

Prostate cancer (PCa) is a common urinary system malignancy in men, ranking the second in the causes of cancer-related deaths [1, 2]. Due to the lack of early symptoms, most local or distant metastases already exist at the time of diagnosis. Relevant data showed that the 5-year survival rate of patients with early local PCa is close to 100%, but it will drop to below 30% for patients with distant metastasis [3]. Surgery and radiotherapy are the main treatment methods for early prostate cancer and achieve good therapeutic effects [4]. For advanced PCa, androgen deprivation therapy (ADT) is currently the main treatment method, including surgical castration or drug castration [5]. However, most patients will eventually develop castration-resistant prostate cancer (CRPC) with poor prognosis [6]. Docetaxel (DTX) is a semisynthetic broad-spectrum paclitaxel antitumor drug for a variety of cancers, including PCa, gastric cancer, liver cancer, breast cancer, and ovarian cancer. Docetaxel- (DTX-) based chemotherapy is the preferred treatment for CRPC in the guidelines [7]. However, high-dose use of DTX will cause patients to have unbearable adverse reactions [8]. Therefore, it is necessary to find new drug combinations for the treatment of CRPC.

Oncolytic adenovirus can specifically infect, proliferate, and lyse tumor cells [9]. The virus released after cell lysis can spread to local or distant metastatic tumor tissues, producing amplification effect. The modified oncolytic adenovirus can provide multiple cloning sites for the insertion of therapeutic genes. Sperm-associated antigen 9 (SPAG9) gene encodes 766 amino acids and can participate in the MAPK signaling pathway through JNK structural homology to regulate cell activity [10]. SPAG9 is highly expressed in various malignant tumor tissues such as PCa, renal cancer, breast cancer, bladder cancer, and lung cancer [11–15]. Silencing the expression of SPAG9 in triple-negative breast cancer and liver cancer effectively inhibited the proliferation and migration of tumor cells [16, 17].

Differential display code 3 (DD3) is a noncoding gene highly expressed in PCa tissues with little or no expression in normal prostate tissues or other tumor tissues [18]. The specificity and sensitivity of DD3 is higher than traditional PCa markers [19]. DD3 promoter is a PCa-specific promoter and restricts specific replication and propagation of oncolytic adenovirus in PCa, improving the safety and targeting of oncolytic adenovirus therapy.

In this study, we used DD3 promoter specificity to construct oncolytic adenovirus armed with SPAG9 shRNA to specially knockdown SPAG9 in PCa cells and examined whether constructed DD3-ZD55-SPAG9 improved the efficacy of DTX for PCa treatment in vivo and in vitro.

## 2. Materials and Methods

### 2.1. Cell Culture

Human prostate cancer cell lines PC-3 and DU-145, human prostatic stromal myofibroblast cell line WPMY-1, and human embryonic kidney 293 cell line HEK-293 were purchased from the Institute of Biochemistry, Chinese Academy of Sciences, and cultured in RPMI-1640 or DMEM (Gibco, MA, USA) supplemented with 10% fetal bovine serum in a humid incubator at 37°C with 5% CO_2_. DTX was provided by Jiangsu Hengrui Pharmaceutical and stored at 4°C.

### 2.2. Construction of Adenovirus

DD3-ZD55 and ZD55-EGFP viruses were provided by Jiangsu Key Laboratory of Cancer Biotherapy. SPAG9 shRNA was designed and inserted into DD3-ZD55 to construct recombinant adenovirus DD3-ZD55-SPAG9 shRNA (named DD3-ZD55-SPAG9 for abbreviation). After extensive amplification in HEK-293 cells, recombinant virus was purified by adenovirus purification mini kit (Biomiga, Inc.) and virus titer was determined by QuickTiterTM adenovirus titer immunoassay kit (Cell Biolabs, San Diego, CA, USA).

### 2.3. CCK-8 Assay

DU-145, PC-3, and WPMY-1 cells were seeded in 96-well plates with 3,000 cells per well. Next, the cells were treated with DTX with different concentration gradients (0, 0.5, 1, 2, 4, and 6 ng/ml), DD3-ZD55-SPAG9 with different titers (0, 1, 10, 20, 50, and 100 MOI), PBS, ZD55-EGFP (10 MOI), DD3-ZD55-SPAG9 10 MOI, ZD55-SPAG9 10 MOI, DTX 2 ng/ml, and 5 MOI DD3-ZD55-SPAG9+1 ng/ml DTX for 24 h, 48 h, 72 h, and 96 h. Cell viability was examined by using Cell Counting kit-8 (CCK-8; Dojindo Molecular Technologies, Inc., Japan). Briefly, 10 *μ*l of CCK-8 was added to each well. After incubation for 2 h, microplate reader was used to measure the absorbance at 450 nm.

### 2.4. Scratch Wound Healing Assay

PC-3 and DU-145 cells were seeded in 6-well plates and grown to 70% confluency. The pipette tip was used to gently scratch the cell monolayer. The plates were then washed with PBS twice to wash off the shed cells and then incubated in medium containing 1% fetal bovine serum. The cells were pictured at 0 and 24 h after scratch under a microscope (Olympus, Japan). The wound healing area (%) was calculated as the distance of the cell-free area at 24 h after scratch relative to that at 0 h after scratch.

### 2.5. Transwell Assay

PC-3 and DU-145 cells were added to the upper chamber of the Transwell (Corning Costar; Oneonta, USA), while the lower chamber was filled with 600 *μ*l of RPMI 1640 medium containing 10% fetal bovine serum. After incubation for 24 h, the cells were fixed with 95% methanol, stained with crystal violet for 20 min, and photographed under the microscope to count the number of invaded cells.

### 2.6. Hoechst 33258 Staining

Hoechst 33258 staining was used to detect cell apoptosis. PC-3 and DU-145 cells were seeded in 6-well plates, fixed with 4% paraformaldehyde, stained with Hoechst 33258 for 10 min, and washed with PBS three times. The cells were photographed under a fluorescence microscope to count the number of apoptotic cells.

### 2.7. Western Blot Analysis

The tumor cells and transplanted tumor tissues were lysed on ice in RIPA buffer (Biyuntian, Shanghai, China) containing 1% benzylsulfonyl fluoride (PMSF) (Solaibao, Beijing, China). The lysates were separated by SDS-PAGE and transferred to the membranes. The membranes were then incubated with the antibodies for SPAG91 (1 : 1000, Abcam, UK), E-cadherin (1 : 1000, Abcam, UK), MMP-2 (1 : 1000, Abcam, UK), Vimentin (1 : 1000, Abcam, UK), and GAPDH (1 : 1000, Abcam, UK) overnight at 4°C and then incubated with secondary antibody at room temperature for 2 h. Then, the protein band was detected with ECL reagent, and the gray value of the band was analyzed by ImageJ software.

### 2.8. Animal Experiments

Male BALB/c nude mice (4-6 weeks old) were provided by Cyper-Bikai Laboratory Animal Co., Ltd. (Shanghai, China), and all animal experimental procedures were approved by Committee for Care and Use of Laboratory Animals of Xuzhou Medical University. A xenograft tumor model was established by subcutaneously injecting 1 × 10^6^ PC-3 cells into the back of the right thigh of each mouse. The mice were randomly divided into 5 groups (*n* = 5): (1) PBS group: no treatment; (2) DTX group: weekly intraperitoneal injection of DTX 10 mg/kg; (3) DD3-ZD55-SPAG9 group: intratumoral injection of DD3-ZD55-SPAG9 (1 × 10^8^ pfu) every 3 days; (4) ZD55-SPAG9 group: intratumoral injection of ZD55-SPAG9 (1 × 10^8^ pfu) every 3 days; (5) DD3-ZD55-SPAG9+DTX group: DTX (5 mg/kg) was injected intraperitoneally every week, and ZD55-SPAG9 (5 × 10^7^ pfu) was intraperitoneally injected every 3 days. The tumor volume was measured on the 7th day after the cells were seeded with PC-3 cells. Then, the tumor volume was measured every 7 days. After 28 days, all nude mice were sacrificed. Tumor volume (TV) calculation formula TV (mm^3^) = length × width^2^ × 0.5.

### 2.9. Immunohistochemical Staining

The tumor sections were dissected from mice, fixed in paraffin wax, and cut into sections of 5 *μ*m thick. The sections were blocked with endogenous peroxidase activity with 3% hydrogen peroxide. After 30 min incubation with blocking serum, sections were incubated with antibodies for SPAG9 (1 : 200, Abcam, UK), E-cadherin (1 : 500, Abcam, UK), MMP-2 (1 : 500, Abcam, UK), and Vimentin (1 : 500, Abcam, UK) overnight at 4°C. The sections were stained by DBA kit (ZSGB-Bio, Beijing, China) and observed with light microscope (Nikon DS-Ri1, Japan). The sections were also counterstained with hematoxylin-eosin. ImageJ software was used to analyze the score of immunohistochemical staining which was expressed as mean density.

### 2.10. TUNEL Assay

The deoxyribonucleotide terminal transferase-mediated nick end labeling kit (TUNEL, KGI, Nanjing, China) was used to detect apoptotic cells in tumor tissues. Formalin-fixed, paraffin-embedded tumor samples were cut into sections and treated with proteinase K for 15 min. The sections were incubated with terminal deoxynucleotidyl transferase (TdT) and FITC-labeled streptavidin. The nuclei were stained with DAPI and observed under fluorescence microscope (Olympus, Japan).

### 2.11. Statistical Analysis

Data were expressed as mean ± SD and analyzed by SPSS 22.0 software. *T*-test was employed for the comparison between two groups. One-way ANOVA was employed for the comparison of multiple groups. *P* <0.05 was considered significant.

## 3. Results

### 3.1. DD3-ZD55-SPAG9 Combined with DTX Inhibited PCa Cell Proliferation

**Figure 1:**
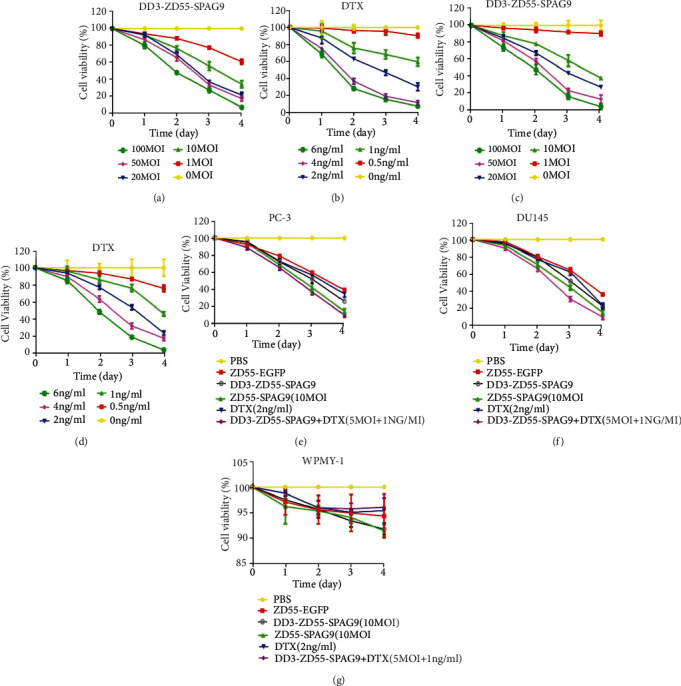
The cytotoxicity of DD3-ZD55-SPAG9 on prostate cancer and normal prostate cells. (a) Different titers of DD3-ZD55-SPAG9 inhibited the proliferation of PC-3 cells. (b) Different concentration gradient of DTX inhibited the proliferation of PC-3 cells. (c) Different titers of DD3-ZD55-SPAG9 inhibited the proliferation of DU-145 cells. (d) Different concentration gradient of DTX inhibited the proliferation of DU-145 cells. (e) Different combinations of treatment inhibited the proliferation of PC-3 cells. (f) Different combinations of treatment inhibited the proliferation of DU-145 cells. (g) CCK-8 assay indicated the inhibition of WPMY-1 cell proliferation by different treatments. Data were expressed as mean ± SD (*n* = 3).

CCK-8 assay showed that DD3-ZD55-SPAG9 inhibited the proliferation of PC-3 and DU-145 cells in time- and concentration-dependent manner. After 48 hours of treatment with 10 MOI DD3-ZD55-SPAG9, the viability of PC-3 cells decreased significantly compared to PBS group (Figure 1(a)). DTX inhibited the proliferation of PC-3 in a time- and concentration-dependent manner (Figure 1(b)). Similar results were observed in DU-145 cells (Figures 1(c) and 1(d)). Furthermore, the combined use of DD3-ZD55-SPAG9 with DTX effectively inhibited the proliferation of PC-3 and DU-145 cells, with significant difference compared to other groups (Figures 1(e) and 1(f)).

To demonstrate that DD3-ZD55-SPAG9 has cytotoxicity only in prostate cancer cells, we performed CCK-8 assay on prostatic stromal myofibroblast WPMY-1 cells as the control. The results showed that the combined treatment of 5 MOI DD3-ZD55-SPAG9 and 1 ng/ml DTX had little cytotoxicity on WPMY-1 cells (Figure 1(g)).

### 3.2. DD3-ZD55-SPAG9 Combined with DTX Inhibited Migration and Invasion of PCa Cells

**Figure 2:**
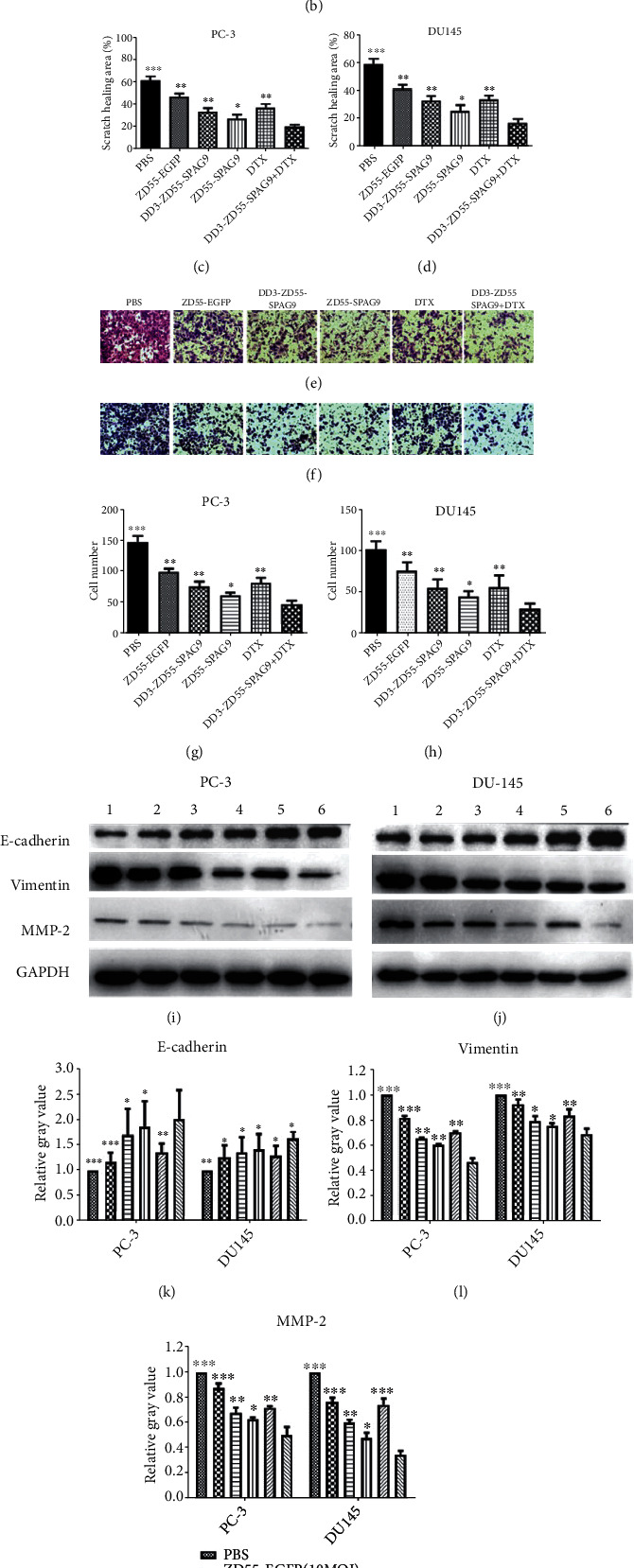
Combination treatment inhibited PCa cell migration and invasion. (a) The scratch wound healing assay of the migration of PC-3 cells (100x). (b) The scratch wound healing assay of the migration of DU-145 cells (100x). (c) Histogram of wound healing area (%) of different treatment groups of PC-3 cells. (d) Histogram of wound healing area (%) of different treatment groups of DU-145 cells. (e) Transwell assay of the invasion of PC-3 cells (200x). (f) Transwell assay of the invasion of DU-145 cells (200x). (g) Histogram of the number of invasion cells of different treatment groups of PC-3 cells. (h) Histogram of the number of invasion cells of different treatment groups of DU-145 cells. (i) Western blot analysis of E-cadherin, vimentin, and MMP-2 in PC-3 cells. (j) Western blot analysis of E-cadherin, vimentin, and MMP-2 in DU-145 cells. (k) Quantitative analysis of E-cadherin levels in PC-3 and DU-145 cells. (l) Quantitative analysis of vimentin levels in PC-3 and DU-145 cells. (m) Quantitative analysis of MMP-2 levels in PC-3 and DU-145 cells. (1) PBS, (2) ZD55-EGFP, (3) DD3-ZD55-SPAG9, (4) ZD55-SPAG9, (5) DTX, and (6) DD3-ZD55-SPAG9+DTX. Data were expressed as mean ± SD (*n* = 3). ^∗^*p* < 0.05, ^∗∗^*p* < 0.01, and ^∗∗∗^*p* < 0.001 compared to the DD3-ZD55-SPAG9+DTX group.

Wound healing assay showed that in PC-3 cells the percentage of scratch healing in DD3-ZD55-SPAG9+DTX, DTX, ZD55-SPAG9, DD3-ZD55-SPAG9, ZD55-EGFP, and PBS groups were 20.03 ± 2.02%, 37.07 ± 3.17%, 27.63 ± 3.24%, 33.97 ± 2.56%, 46.60 ± 2.85%, and 64.13 ± 3.48%, respectively. In DU-145 cells, the percentage of scratch healing in DD3-ZD55-SPAG9+DTX, DTX, ZD55-SPAG9, DD3-ZD55-SPAG9, ZD55-EGFP, and PBS groups were 17.53 ± 2.73%, 33.97 ± 2.96%, 25.63 ± 4.33%, 33.07 ± 3.46%, 41.60 ± 2.74%, and 59.13 ± 3.62%, respectively. The healing area of the scratches in each treatment group was lower than that in the PBS group, and the wound healing area of the combined group was the lowest (Figures 2(a)–2(d)). These results suggested that combined use of DD3-ZD55-SPAG9 and DTX effectively inhibited the migration ability of PCa cells.

Transwell assay showed that in PC-3 cells, the number of invasive cells in DD3-ZD55-SPAG9+DTX, DTX, ZD55-SPAG9, DD3-ZD55-SPAG9, ZD55-EGFP, and PBS groups was 49.25 ± 5.09, 82.88 ± 8.51, 62.63 ± 4.27, 76.88 ± 8.20, 100.63 ± 5.29, and 149.13 ± 9.01, respectively. In DU-145 cells, the number of invasive cells in DD3-ZD55-SPAG9+DTX, DTX, ZD55-SPAG9, DD3-ZD55-SPAG9, ZD55-EGFP, and PBS groups was 31.33 ± 6.16, 57.00 ± 14.47, 45.00 ± 6.93, 55.89 ± 10.40, 76.00 ± 10.94, and 102.88 ± 9.30, respectively. The number of invasive cells in each treatment group was lower than that in the PBS group, and the number of invasive cells in the combination group was the lowest (Figures 2(e)–2(h)). These results suggested that combined use of DD3-ZD55-SPAG9 and DTX effectively inhibited the invasion ability of PCa cells.

Because cancer invasion is related to epithelial to mesenchymal transition (EMT), we detected the expression of EMT-related proteins. Western blot analysis showed that compared with PBS group, the expression of E-cadherin increased while the expression of vimentin and MMP-2 decreased in each treatment group of PC-3 and DU-145 cells. In the DD3-ZD55-SPAG9+DTX group, the expression of E-cadherin was the highest, and the expression of vimentin and MMP-2 was the lowest (Figures 2(i)–2(m)). These data indicated that combined treatment effectively inhibited the EMT process of PCa.

### 3.3. DD3-ZD55-SPAG9 Combined with DTX Induced Apoptosis of PCa Cells In Vitro

**Figure 3:**
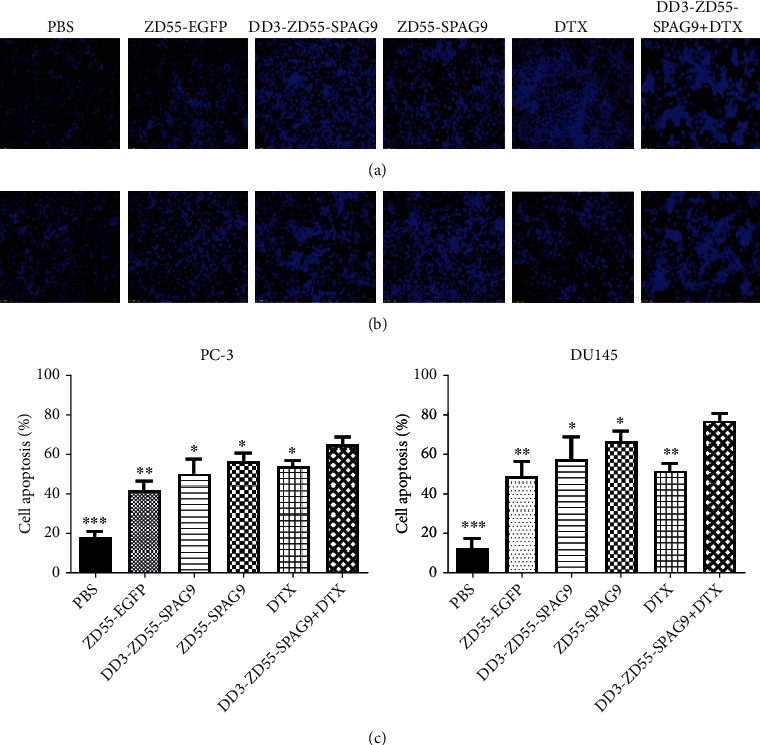
Combined treatment promoted PCa cell apoptosis. (a) Hoechst 33258 staining of PC-3 cells (200x). (b) Hoechst 33258 staining of DU-145 cells. (c) Histogram of apoptosis rate of different treatment groups of PC-3 and DU-145 cells. Data were expressed as mean ± SD (*n* = 3). ^∗^*p* < 0.05, ^∗∗^*p* < 0.01, and ^∗∗∗^*p* < 0.001 compared to the DD3-ZD55-SPAG9+DTX group.

Hoechst 33258 staining showed that the apoptosis rate in PC-3 cells of DD3-ZD55-SPAG9+DTX, DTX, ZD55-SPAG9, DD3-ZD55-SSPAG9, ZD55-EGFP, and PBS groups was 77.17 ± 3.30, 51.71 ± 3.84, 66.52 ± 5.30, 57.57 ± 5.57, 43.79 ± 7.74, and 12.84 ± 4.59, respectively, and the apoptosis rates in DU145 cells of DD3-ZD55-SPAG9+DTX, DTX, ZD55-SPAG9, DD3-ZD55-SSPAG9, ZD55-EGFP, and PBS groups was 64.75 ± 4.28, 54.21 ± 2.93, 56.85 ± 4.37), 50.15 ± 8.16, 42.16 ± 4.47, and 18.37 ± 3.00, respectively. The apoptosis rate of the treatment group was higher than that in the PBS group. Compared with the single treatment group, the DD3-ZD55-SPAG9+DTX group had significantly higher apoptosis rate (Figures 3(a)–3(c)).

### 3.4. DD3-ZD55-SPAG9 Combined with DTX Inhibited the Growth of Xenograft Tumor

**Figure 4:**
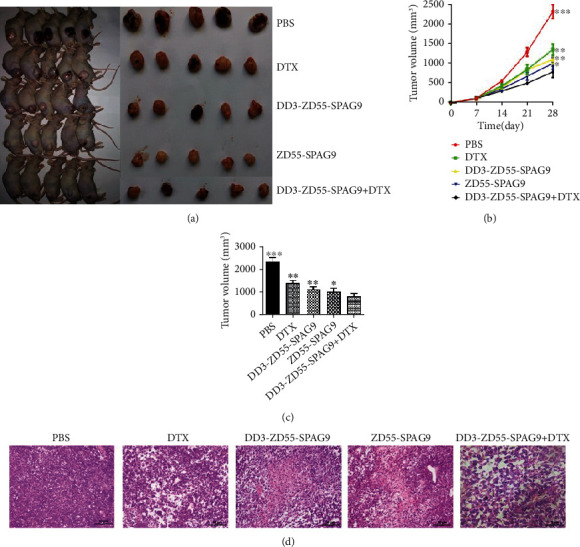
Combination therapy inhibits xenograft tumor growth. (a) Pictures of nude mice and each groups of xenograft tumors. (b) Growth curve of xenograft tumors in each group. (c) Histogram of tumor volume of each group on 28th day. Data were expressed as mean ± SD (*n* = 5). ^∗^*p* < 0.05, ^∗∗^*p* < 0.01, and ^∗∗∗^*p* < 0.001 compared to the DD3-ZD55-SPAG9+DTX group. (d) HE staining of xenografts of different groups (400x).

We established the PC-3 cell subcutaneous xenograft tumor model to verify the therapeutic effect in vivo. The tumor growth rate was measured, and a volume measurement was performed every 7 days. After 28 days, all nude mice were sacrificed by neck dissection and all xenograft tumors were dissected. The results showed that at 28 days tumor volumes of the DD3-ZD55-SPAG9, ZD55-SPAG9, DD3-ZD55-SPAG9, DTX, and PBS groups were 784.02 ± 145.59, 1003.21 ± 150.89, 1104.04 ± 110.39, 1352.00 ± 142.57, and 2321.36 ± 173.88. The tumor volume of the treatment group was lower than that of the PBS group, and the tumor volume of the DD3-ZD55-SPAG9+DTX group was the smallest (Figures 4(a)–4(c)). HE staining of the paraffin sections of the tumors in each group showed that the cells in the PBS group had different sizes and disordered arrangement, the nucleolus had obvious division phenomena, and the chromatin staining was deep, with typical atypia. In each treatment group, there were more necrotic cells, shrinkage of cell membranes, cell lysis, accompanied by nuclear shrinkage and fragmentation. Compared with the single treatment group, the DD3-ZD55-SPAG9+DTX group had more cell lysis and nuclear shrinkage and fragmentation (Figure 4(d)). These results indicated that combined use of DD3-ZD55-SPAG9 and DTX effectively inhibited the growth of subcutaneous xenografts in nude mice.

### 3.5. DD3-ZD55-SPAG9 Combined with DTX Induced Apoptosis of PCa In Vivo

**Figure 5:**
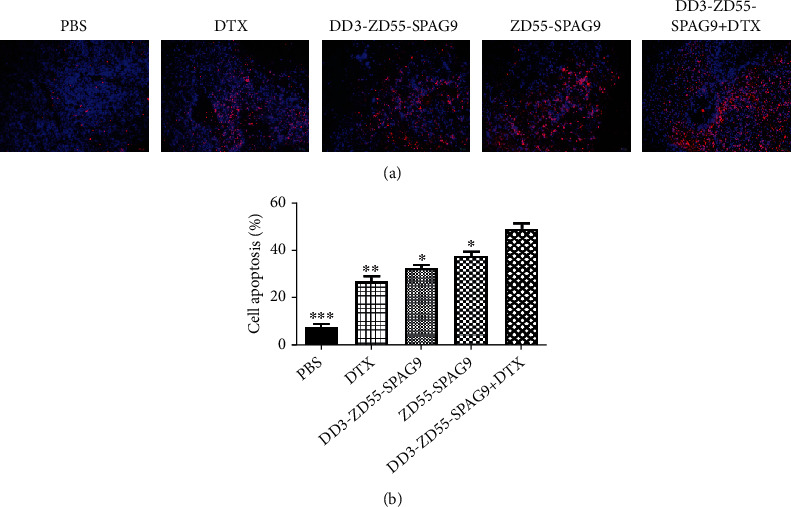
Combination therapy promoted tumor cell apoptosis in vivo. (a)TUNEL staining of xenografts of different groups (400x). (b) Histogram of apoptosis rate of each group. Data were expressed as mean ± SD (*n* = 3). ^∗^*p* < 0.05, ^∗∗^*p* < 0.01, and ^∗∗∗^*p* < 0.001 compared to the DD3-ZD55-SPAG9+DTX group.

Next, we detected the apoptosis of xenograft tumor cells in each group by TUNEL. The results showed that compared with the PBS group, there were more apoptotic cells in the DD3-ZD55-SPAG9+DTX, ZD55-SPAG9, DD3-ZD55-SPAG9, and DTX groups. The apoptosis rates of DD3-ZD55-SPAG9+DTX, ZD55-SPAG9, DD3-ZD55-SPAG9, DTX, and PBS groups were 44.86 ± 3.01%, 37.47 ± 1.88%, 32.56 ± 1.37%, 26.74 ± 2.45%, and 7.31 ± 1.42%, respectively. Compared with the single treatment group, the apoptosis rate was significantly higher in the combined group (Figures 5(a) and 5(b)). These results illustrated that the combined use of DD3-ZD55-SPAG9 and DTX could effectively promote apoptosis in tumor cells.

### 3.6. DD3-ZD55-SPAG9 Combined with DTX Regulated the Expression of E-Cadherin, Vimentin, and MMP-2 Proteins in Xenograft Tumor

**Figure 6:**
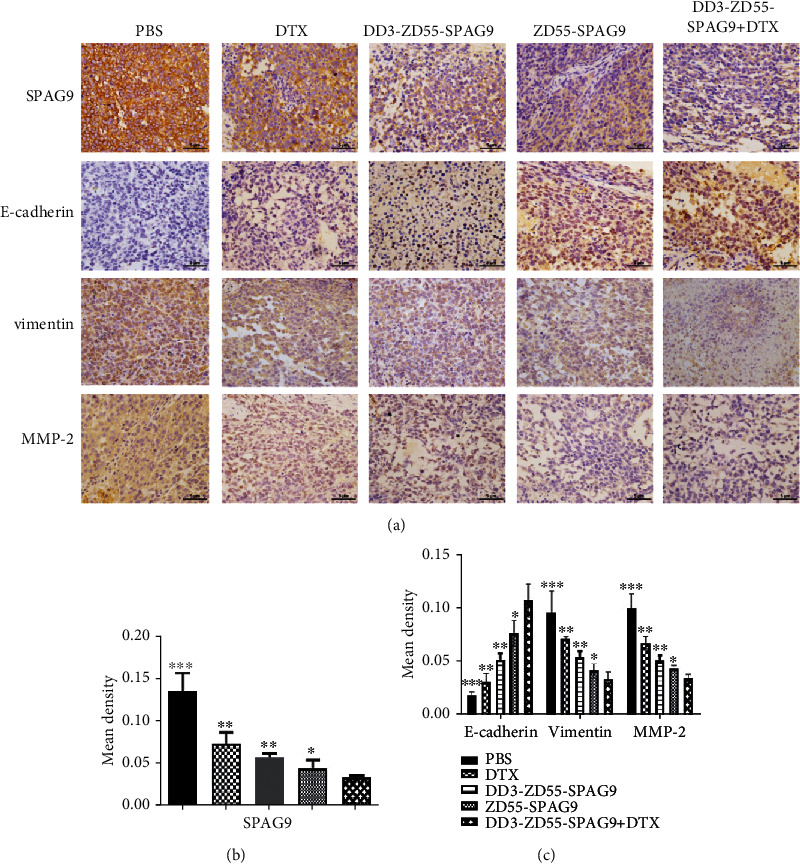
Combined therapy regulated EMT-related protein expression. (a) Immunohistochemistry staining of SPAG9, E-cadherin, vimentin, and MMP-2 in xenografts of different groups (400x). (b) Analysis of SPAG9 staining density for each group. (c) Analysis of E-cadherin, vimentin, and MMP-2 staining density for each group. ^∗^*p* < 0.05, ^∗∗^*p* < 0.01, and ^∗∗∗^*p* < 0.001 compared to the DD3-ZD55-SPAG9+DTX group.

Immunohistochemical staining showed that the expression of SPAG9, vimentin, and MMP-2 was lower while the expression of E-cadherin was higher in each treatment group compared to the PBS group (Figure 6(a)). Quantitative analysis of optical density of SPAG9, E-cadherin, vimentin, and MMP-2 in DD3-ZD55-SPAG9+DTX, ZD55-SPAG9, DD3-ZD55-SPAG9, DTX, and PBS groups showed that the combined treatment group had the lowest SPAG9, vimentin, and MMP-2 protein expressions and the highest E-cadherin protein expression (Figures 6(b) and 6(c)). These results suggested that the combination therapy may inhibit tumor EMT in vivo.

## 4. Discussion

DD3 is one of the most prostate cancer-specific genes with high expression in prostate cancer tissues but no expression in prostate cancer adjacent tissues, benign prostatic hyperplasia, or normal prostate tissues [20]. Therefore, in this study, we constructed a recombinant oncolytic adenovirus DD3-ZD55-SPAG9 to specifically regulate the replication of adenovirus in PCa cells through DD3. Our results showed that DD3-ZD55-SPAG9 effectively silenced SPAG9; inhibited PCa cell proliferation, migration and invasion; and induced PCa cell apoptosis. Moreover, compared with ZD55-SPAG9, DD3-ZD55-SPAG9 exhibited significantly lower toxic effect on normal prostate cells.

DTX is the first-line chemotherapy drug recommended for PCa, but it has toxicity and side effect on patients. In this study, we reduced the dosage of DTX and combined with DD3-ZD55-SPAG9 to explore antitumor effects in vitro and in vivo. The results showed that DD3-ZD55-SPAG9+DTX had better effects than single treatment in inhibiting PCa cell proliferation, migration, and invasion and inducing PCa cell apoptosis.

EMT indicates the transformation of epithelial cells into mesenchymal cells and is one of the common mechanisms of tumor metastasis [21]. During EMT, there will be changes in the expression of various proteins, such as decreased expression of E-cadherin and increased expression of N-cadherin and Vimentin [22–24]. MMP family plays an important role in the process of tumor metastasis [25]. MMP-2 can digest tumor extracellular matrix, creating a pathway for tumor cell metastasis [26]. In this study we found that DD3-ZD55-SPAG9+DTX had better effects than single treatment in inhibiting SPAG9, vimentin, and MMP-2 expressions, and promoting E-cadherin expression in PCa in vitro and in vivo.

Promoting the apoptosis of tumor cells is one of the antitumor mechanisms of chemotherapy [27]. Consistently, in this study, we found that while DTX treatment alone promoted PCa cell apoptosis both in vitro and in vivo, the combined use of DD3-ZD55-SPAG9 and DTX significantly enhanced PCa cell apoptosis both in vitro and in vivo. These results suggest that the superior antitumor efficacy of DD3-ZD55-SPAG9 may be mediated by inducing PCa cell apoptosis.

In summary, we constructed oncolytic adenovirus DD3-ZD55-SPAG9 driven by DD3 promoter for targeted therapy of PCa. DD3-ZD55-SPAG9 exhibited superior efficacy and specificity to kill PCa cells both in vitro and in vivo without toxicity on normal prostate cells, which may be related to the induction of PCa cell apoptosis and the inhibition of EMT of PCa. DD3-ZD55-SPAG9 has promising application to enhance chemotherapy of PCa.

## Data Availability

All data are included in this manuscript.
